# Supplementary figures and images for: A comparison of short-read, HiFi long-read, and hybrid strategies for genome-resolved metagenomics
Source: Microbiol Spectr. 2024 Mar 7;12(4):e03590-23. doi: 10.1128/spectrum.03590-23 (PMC10986573; doi:10.1128/spectrum.03590-23)

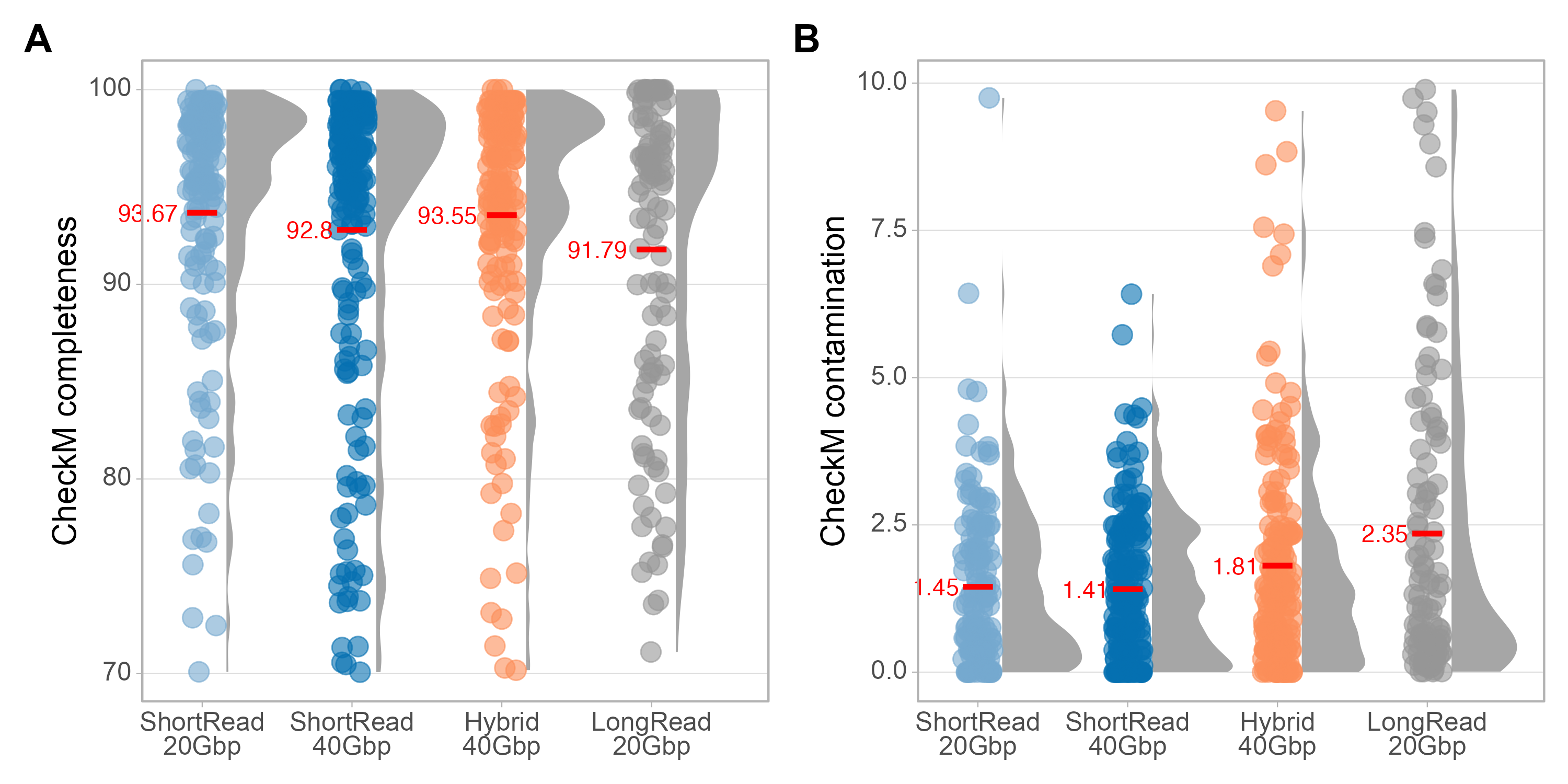

Supplement: Figure S1 — CheckM completeness and contamination scores for the metagenome assembled genomes. [file spectrum.03590-23-s0001.tiff]

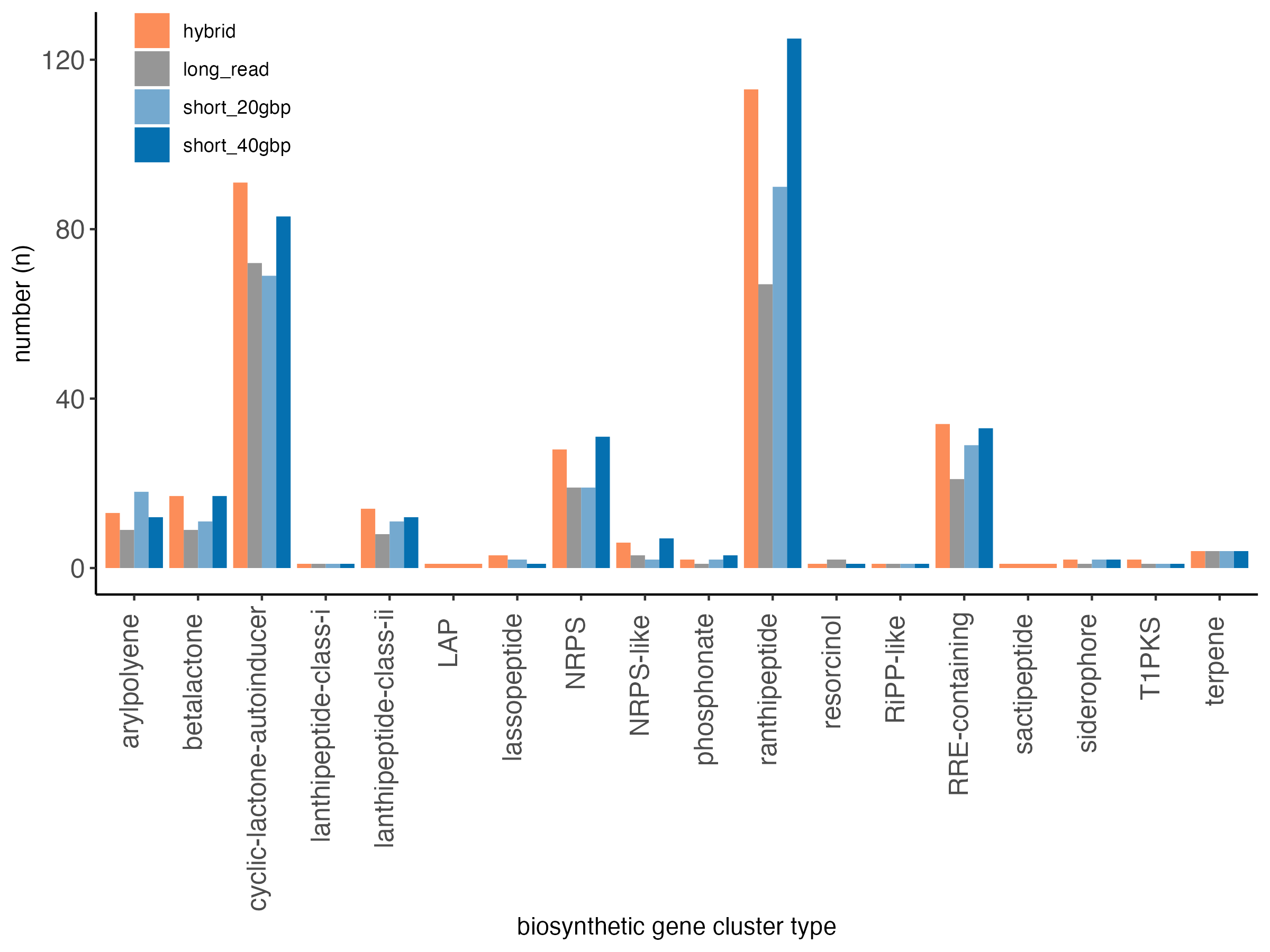

Supplement: Figure S2 — Counts of different biosynthetic gene clusters predicted by antiSMASH. [file spectrum.03590-23-s0002.tiff]

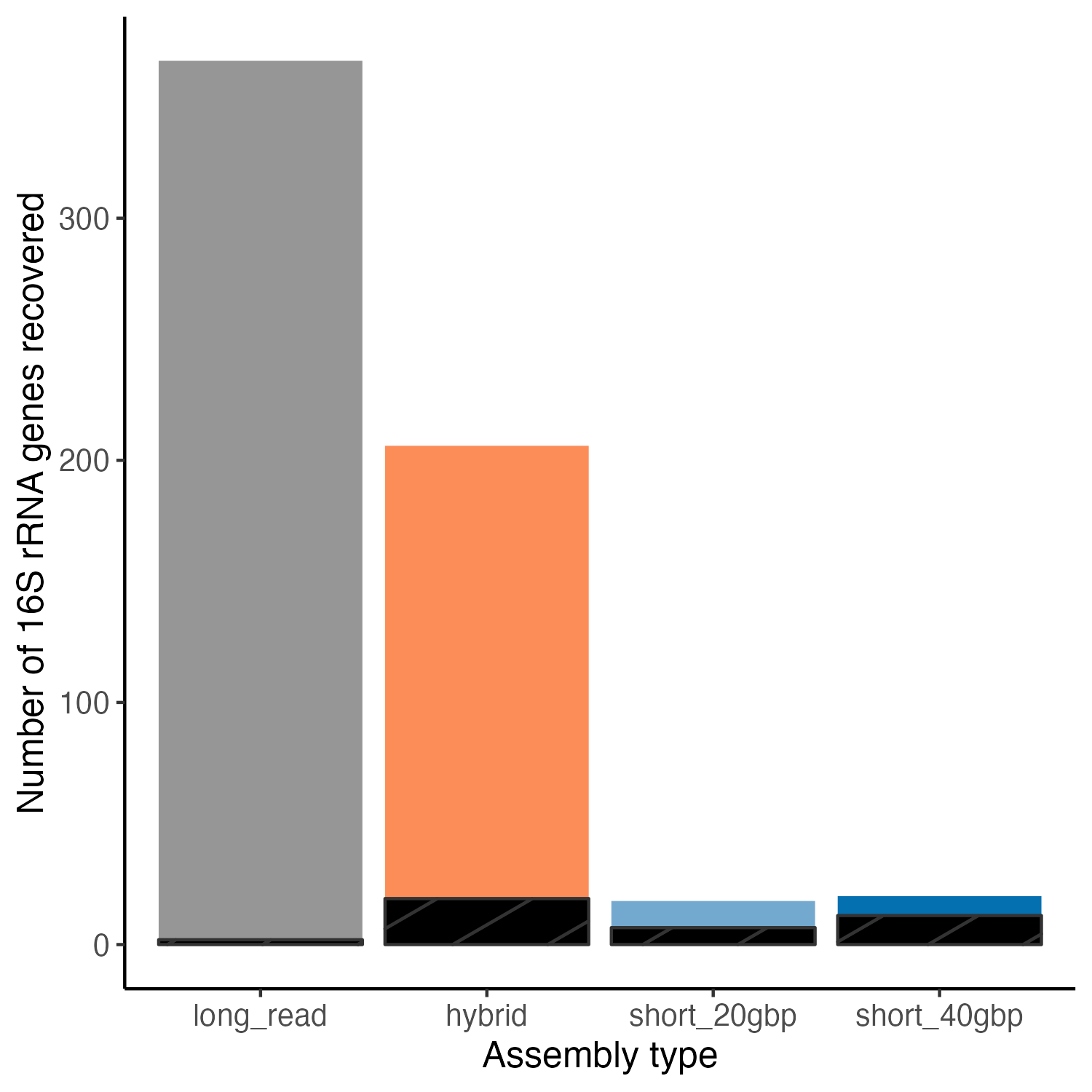

Supplement: Figure S3 — Number of 16S rRNA genes recovered by different assembly types. [file spectrum.03590-23-s0003.tiff]
