# Supplementary material for: A comparison of short-read, HiFi long-read, and hybrid strategies for genome-resolved metagenomics
Source: Microbiol Spectr. 2024 Mar 7;12(4):e03590-23. doi: 10.1128/spectrum.03590-23 (PMC10986573; doi:10.1128/spectrum.03590-23)
Supplement: Legends — for the supplemental figures. [file spectrum.03590-23-s0005.docx]

# Supplementary figures

**SI figure 1 |** CheckM completeness (A) and contamination (B) scores for the metagenome assembled genomes.

**SI figure 2 |** Counts of different biosynthetic gene clusters predicted by antiSMASH.

**SI figure 3 |** Number of 16S rRNA genes recovered by different assembly types. Black bars indicate incomplete 16S rRNA genes (<1,450 bp length).

**SI figure 4 |** Summary of circular contigs extraction from each dataset type annotated by viralVerify. A) Total number of circular contigs identified. B) Total length in megabase pairs (Mbp) of circular contigs.
